# Orbital Delocalization and Enhancement of Magnetic Interactions in Perovskite Oxyhydrides

**DOI:** 10.1038/srep19653

**Published:** 2016-01-25

**Authors:** Kai Liu, Yusheng Hou, Xingao Gong, Hongjun Xiang

**Affiliations:** 1 Key Laboratory of Computational Physical Sciences (Ministry of Education), State Key Laboratory of Surface Physics, Collaborative Innovation Center of Advanced Microstructures, and Department of Physics, Fudan University, Shanghai 200433, P.R. China

## Abstract

Recent experiments showed that some perovskite oxyhydrides have surprisingly high magnetic-transition temperature. In order to unveil the origin of this interesting phenomenon, we investigate the magnetism in SrCrO_2_H and SrVO_2_H on the basis of first-principles calculations and Monte Carlo simulations. Our work indicates that the Cr-O-Cr superexchange interaction in SrCrO_2_H is unexpectedly strong. Different from the previous explanation in terms of the H^−^ ion substitution induced increase of the Cr-O-Cr bond angle, we reveal instead that this is mainly because the 3*d* orbitals in perovskite oxyhydrides becomes more delocalized since H^−^ ions have weaker electronegativity and less electrons than O^2−^ ions. The delocalized 3*d* orbitals result in stronger Cr-O interactions and enhance the magnetic-transition temperature. This novel mechanism is also applicable to the case of SrVO_2_H. Furthermore, we predict that SrFeO_2_H will have unprecedented high Neel temperature because of the extraordinarily strong Fe-H-Fe σ-type interactions. Our work suggests the anion substitution can be used to effectively manipulate the magnetic properties of perovskite compounds.

Complex transition metal oxides have been the subject of enduring interest due to the wide variety of physical properties they exhibit, to name a few, high-*T*_c_ superconductivity, magnetoresistance, multiferroicity, thermoelectric response, and so on^1^,^2^,^3^,^4^,^5^,^6^,^7^. For the past few years, scientists have found that replacing segmental O^2−^ ions in transition metal oxides by N^3−^, F^−^ or S^2−^ can result in novel materials such as pigments, water-splitting photocatalysis, dielectric and cathode material^8^,^9^,^10^,^11^,^12^. Different from N^3−^, F^−^ or S^2−^ which has *p* valence electrons, the H^−^ anion has a filled 1 *s*^2^ electronic configuration that is fundamentally different from the O^2−^ ion case. Therefore, it is expected that the H^−^ ion substitution might leads to exotic behaviors in perovskite. In pioneering works, a large amount of hydrogen species were incorporated into ATiO_3_ (A = Ba, Sr, Ca) and Sr_2_VO_4_ lattice through the use of CaH_2_ reductant^13^,^14^,^15^,^16^. The resulting oxyhydride ATiO_3−*x*_H_*x*_ exhibits high electronic conductivity and its hydride ions are exchangeable with gaseous hydrogen at elevated temperature, indicating that it can be an ideal mixed electron/hydride proton conductor for electrochemical applications^13^,^14^,^15^. In oxyhydride Sr_2_VO_4−*x*_H_*x*_, the hydride ion could act as an effective carrier dopant because the hydrogen and oxygen concentrations can be controlled^16^.

Interestingly, it was experimentally found that the magnetic properties of transition metal oxides may change dramatically if some oxygen anions are replaced by hydrogen anions. An antiferromagnetic-to-ferromagnetic transition in EuTiO_3–*x*_H_*x*_ induced by hydride substitution was reported, where the ferromagnetism was caused by the Ruderman–Kittel–Kasuya–Yosida (RKKY) interaction between the Eu^2+^ spins mediated by the itinerant Ti 3*d* electrons^17^. LaSrCoO_3_H_0.7_^18^,^19^,^20^ and Sr_3_Co_2_O_4.33_H_0.84_^21^ were found to display high magnetic transition temperatures. Recently, the stoichiometric perovskite oxyhydrides SrCrO_2_H and SrVO_2_H have been synthesized^22^,^23^. The average structure of SrCrO_2_H is the cubic perovskite where the hydride ions are randomly distributed. In SrVO_2_H, the planar VO_2_ layers are connected by hydride ions. The experimentally observed antiferromagnetic (AFM) Neel temperature (*T*_*N*_) of SrCrO_2_H and SrVO_2_H are around 380 K and higher than 300 K^22^,^23^, respectively. It is puzzling that the Neel temperature *T*_*N*_ of SrCrO_2_H is higher than that (290 K)^24^,^25^ of LaCrO_3_. In both SrCrO_2_H and LaCrO_3_, the valence of Cr element is 3+ with the three 3*d* electrons occupying the *t*_2g_ orbitals, that is, the electron configuration is (*d*_*xy*_)^1^(*d*_*yz*_)^1^(*d*_*xz*_)^1^. In average, each Cr^3+^ ion in SrCrO_2_H has two Cr-H-Cr superexchange paths and four Cr-O-Cr superexchange paths. Since the *t*_2g_ orbitals of the Cr^3+^ ion could not interact with the 1s orbitals of H^−^ ions by symmetry, the Cr-H-Cr superexchange interaction is negligible. In LaCrO_3_, there are six Cr-O-Cr superexchange paths for each Cr^3+^ ion. So the fact that the Neel temperature *T*_*N*_ of SrCrO_2_H is higher than that of LaCrO_3_ is rather unexpected.

To probe the origin of the high *T*_*N*_ in SrCrO_2_H and SrVO_2_H, we studied the magnetic properties of SrCrO_2_H, SrVO_2_H, and LaCrO_3_ based on the density functional theory (DFT). We show that due to weaker electronegativity and less electrons of H^−^ ions than those of O^2−^ ions, the substitution of H^−^ ions with O^2−^ ions lead to more delocalized 3*d* orbitals of Cr^3+^ ions which make Cr-O-Cr superexchange in SrCrO_2_H much stronger and leads to a high *T*_*N*_. This new mechanism is also applicable to the case of SrVO_2_H.

Although the H^−^ ion was experimentally reported to be randomly distributed in SrCrO_2_H, we will adopt an ordered structure (see Fig. 1a) to investigate the magnetism in SrCrO_2_H for the following reasons. First, by using the cluster expansion approach, we find the ground state structure of SrCrO_2_H is similar to the experimentally observed structure of SrVO_2_H. Second, we find that the magnetic properties of another SrCrO_2_H structure with a more random H^−^ ion distribution are similar to those of the ordered structure (see Supplementary Material). The ground state structure (tetragonal *P4/mmm* symmetry) of SrCrO_2_H has one H^−^ ion and two O^2−^ ions in the primitive cell. The Cr^3+^ cations are located within square planes of oxide ions and form CrO_2_ sheets. The CrO_2_ sheets are connected by hydride ions, which occupy the remaining two coordination sites around each Cr^3+^ center. Thus the ground state structure of SrCrO_2_H can be described by a CrO_2_-SrH-CrO_2_-SrH stacking sequence. The computed phonon frequencies^26^ (see Supplementary Figure S3) indicate that the ground state structure of SrCrO_2_H is dynamically stable.[Fig f2]

To examine the magnetic properties of SrCrO_2_H, we considered four ordered spin states, namely, the ferromagnetic (FM), A-type AFM, C-type AFM and G-type AFM states. The experimentally observed AFM structure is the G-type. Our GGA + *U* calculations show that all the AFM states have lower energy than the FM state, and the G-type AFM is indeed the ground state, in consistent with experimental observations^22^. To extract the values of the spin exchange parameters, we adopt the four-state mapping approach^27^,^28^. There are four relevant Cr-Cr spin exchange interactions (see Fig. 1a): (1) *J*_O_ is the nearest neighbor (NN) superexchange interaction for the Cr-O-Cr path; (2) *J*_H_ is the NN spin exchange interaction for the Cr-H-Cr path; (3) *J*_3_ is next nearest neighbor (NNN) super-superexchange interaction in the plane; (4) *J*_4_ is the NNN out of plane super-superexchange interaction. The calculated exchange parameters in the tetragonal structure of SrCrO_2_H are summarized in Table 1. As expected, the NN Cr-O-Cr path has the strongest spin exchange interaction (*J*_O_) since it is mediated by the strong π-π hybridization between 3*d* and 2*p* orbitals. The NN spin exchange interaction *J*_H_ is weakly AFM due to the direct through-space overlap between the *t*_2g_ orbitals of Cr^3+^ ions. The NNN exchange interactions (*J*_3_ and *J*_4_) are negligible. The AFM nature of the NN interactions can result in the G-type AFM order. Based on the calculated spin exchange parameters, our Monte Carlo (MC) simulations indicate that the *T*_*N*_ is around 285 K. If a smaller Hubbard U is adopted, we can get higher *T*_*N*_ according to the theory of superexchange, in better agreement with the experimentally observed *T*_*N*_^22^.

For comparison, we also studied the magnetism in LaCrO_3_. Experiments show the ground state structure of LaCrO_3_ is the GdFeO_3_–type distorted perovskite with *Pbnm* space group and its lattice constants are *a* = 5.478, *b* = 7.759, and *c* = 5.516^29^. The lattice constants of our optimized *Pbnm* structure are *a* = 5.468 Å, *b* = 7.758 Å, and *c* = 5.497 Å, in good agreement with experiment. The obtained spin exchange parameters of the optimized LaCrO_3_ are summarized in Table 1. The NN interaction *J*_O_ is AFM and the NNN interactions are negligible. This is in accord with the experimentally observed G-type AFM ground state in LaCrO_3_. Compared with SrCrO_2_H, it is clear that *J*_O_ in LaCrO_3_ is much weaker. Based on the calculated spin exchange parameters, our MC simulations indicate that the *T*_*N*_ for LaCrO_3_ is around 133 K. Therefore, our theoretical calculations confirm the experimental observation that SrCrO_2_H has a higher *T*_*N*_ than LaCrO_3_, as shown in Fig. 3.

Now we begin to understand the difference in the magnetic properties between SrCrO_2_H and LaCrO_3_. There are two significant differences between SrCrO_2_H and LaCrO_3_. First, there is a structural difference. The CrO_6_ octahedron in LaCrO_3_ is tilted due to a small tolerance factor, while there is no octahedron tilt in SrCrO_2_H. As a result, the Cr-O-Cr angle in SrCrO_2_H is 180° while the average Cr-O-Cr angle in LaCrO_3_ is 167°. Second, a chemical difference exists since one third of the O^2−^ ions are replaced by H^−^ ions.

To make clear whether the structural difference or the chemical difference is responsible for the stronger Cr-O-Cr exchange in SrCrO_2_H, we investigate the magnetic properties of SrCrO_2_H and LaCrO_3_ with the same cubic perovskite crystal structure. The lattice constant is set to be the average lattice constant (3.85 Å) of experimental SrCrO_2_H structure^22^. Note that the hypothetic cubic LaCrO_3_ phase can be regarded as a result of substituting Sr^2+^ and H^−^ in cubic SrCrO_2_H with La^3+^ and O^2−^, respectively. The computed spin exchange parameters of these two cubic structures are summarized in Table 1. Surprisingly and interestingly, the Cr-O-Cr superexchange (29.69 meV) in SrCrO_2_H is almost as twice as that (14.25 meV) in LaCrO_3_ despite of the fact that the Cr-O bond length and the Cr-O-Cr bond angle are identical in cubic SrCrO_2_H and LaCrO_3_. As expected, the Cr-O-Cr exchange in cubic LaCrO_3_ is stronger than that (8.88 meV) in *Pbnm* LaCrO_3_ according to the Goodenough-Kanamori rule^30^,^31^,^32^. Previously, it was suggested that the structural difference is solely responsible for the high *T*_*N*_ in SrCrO_2_H^22^. However, our calculations show that the structure difference and chemical difference enhance the Cr-O-Cr exchange interaction by 5.37 meV and 15.44 meV, respectively. Therefore, the effect of chemical difference on the Cr-Cr exchange interaction is much more important than that of the structural difference.

To account for why *J*_O_ in the cubic SrCrO_2_H is as twice as that in the cubic LaCrO_3_, we examine their electronic structures in details. Figure 2a shows the partial density of states (PDOS) of the spin-up Cr^3+^ ion in SrCrO_2_H with the G-type AFM order. We can see that, for SrCrO_2_H, the majority-spin *t*_2g_ (*d*_*xy*_, *d*_*xz*_, and *d*_*yz*_) orbitals are occupied by three electrons but the majority-spin *e*_g_ orbitals are unoccupied, which is similar to the orbital occupation in LaCrO_3_. The energy level and occupancy of the 3*d* orbitals of the tetragonal SrCrO_2_H are shown in Fig. 2b. Note that the *t*_2g_ orbitals split into the low-lying two-fold degenerate *d*_*xz*_/*d*_*yz*_ level and a higher *d*_*xy*_ level as a result of the tetragonal symmetry.

Without loss of generality, we consider the spin exchange *J*_O_ for the Cr1-O-Cr2 path along the *x* axis, as shown in Fig. 1a. According to Anderson’s superexchange theory^33^, the magnitude of the spin exchange can be estimated by 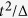, where *t* is the effective hopping between the *d* orbitals and Δ is the energy difference between majority-spin and minority-spin orbitals. In the cubic SrCrO_2_H or LaCrO_3_, the *d*_*xz*_ orbital can only interact with the neighboring *d*_*xz*_ orbital, and so do *d*_*xy*_, *d*_*yz*_. The magnitude of *J*_O_ for the Cr1-O-Cr2 path can be estimated as 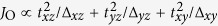, where *t*_m_ (m=*xz, yz, xy*) is effective hopping between the m orbitals of Cr1 and Cr2, and Δ_*m*_ is the energy difference between the majority-spin *m* orbital and minority-spin *m* orbital of the Cr^3+^ ion (see Fig. 2b). The Δ_*m*_ parameters are estimated by constructing the maximally localized Wannier functions (MLWFs) based on the ferromagnetic electronic structure. It turns out that Δ_*m*_ (about 5.3 eV) in SrCrO_2_H is rather close to that (about 5.5 eV) in LaCrO_3_. Therefore, we can regard Δ_*m*_ as a constant. The effective hopping paramters *t*_*m*_ between the Cr 3*d* orbitals is obtained by constructing the MLWFs using the spin-unpolarized Bloch wavefunctions. These hopping parameters are listed in Table 2. We can see that the hopping parameter *t*_*yz*_ between the *d*_*yz*_ orbitals of Cr1 and Cr2 is negligible since these two orbitals are almost parallel to each other. The π-π hopping parameter *t*_*xy*_ in the cubic SrCrO_2_H are the same as that in the cubic LaCrO_3_. The striking result is that the π-π hopping parameter *t*_*xz*_ between the *d*_*xz*_ orbitals of Cr1 and Cr2 in the cubic SrCrO_2_H is almost 50% stronger than that in LaCrO_3_. Using these hopping parameters, we can estimate the ratio between *J*_O_ in cubic SrCrO_2_H and that in LaCrO_3_ as:


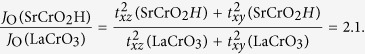


Thus, our first-principles result can be well accounted for by this simple model. This analysis clearly shows that the stronger *t*_*xz*_ hopping in SrCrO_2_H is responsible for its high *T*_*N*_.

Figure 4a,b shows the real-space distribution of the *d*_*xz*_-like MLWFs in the cubic SrCrO_2_H and LaCrO_3_. It is clear that the effective *d*_*xz*_-like MLWF not only distributes around the Cr ion, but also has tails on the neighboring O^2−^ ions due to the anti-bonding π^*^ hybridization between Cr-*d*_*xz*_ and O-2*p* orbitals. It is the tails on the O^2−^ ions that mediate the effective hopping between the Cr-*d*_*xz*_ orbitals. An interesting observation is that the lobes on the O^2−^ ion in the MLWF of SrCrO_2_H are bigger than those in LaCrO_3_. This suggests that the interaction between Cr-*d*_*xz*_ orbital and O-*p*_*z*_ orbital in SrCrO_2_H is stronger than that in LaCrO_3_, in agreement with our previous result that the effective hopping between the *d*_*xz*_ orbitals of Cr1 and Cr2 in SrCrO_2_H is larger than that in LaCrO_3_.

We propose that the stronger interaction between Cr-*d*_*xz*_ orbital and O-*p*_*z*_ orbital in SrCrO_2_H results from the more delocalized Cr-*d*_*xz*_ orbital in SrCrO_2_H (see Supplementary Figure S5). This is supported by a separate MLWF analysis which indicates that the spread of the atomic Cr-*d*_*xz*_ orbital in the cubic SrCrO_2_H is larger than that in the cubic LaCrO_3_. The more delocalized Cr-*d*_*xz*_ orbital in SrCrO_2_H can be reasoned by considering the electrostatic potential exerted on the Cr 3*d* electrons. The contour plots of the electrostatic potential on the *x*-*z* plane are displayed in the Fig. 4c,d. We can see that the electrostatic potential along the Cr-H direction is much weaker than that along the Cr-O direction. Thus, the *d*_*xz*_ orbital in SrCrO_2_H is more delocalized along the *z*-axis, as can also be seen from Fig. 4a. It is the weaker repulsion between the H^−^ ion and the 3*d* electrons that makes the *d*_*xz*_ orbital in SrCrO_2_H more delocalized. The weaker electrostatic potential along the Cr-H direction results from the fact that H^−^ ions have the weaker electronegativity and less charge than O^2−^ ions. Therefore, the replacement of O^2−^ ions by H^−^ ions will not only change the hybridization type between the transition metal and the anions, but also affect the wavefunction distribution of *d* orbitals. Such novel mechanism revealed here for SrCrO_2_H can be also applied to SrVO_2_H. The only difference is that there is one *d* electron less than that in SrCrO_2_H, which makes the *T*_*N*_ slightly lower (see Supplementary Material). We note that first-principles calculations^34^ were recently carried out to study the electronic and magnetic properties of SrVO_2_H. However, the mechanism for the high Neel temperature in SrVO_2_H was not discovered.

The mechanism that the H^−^ ion induced delocalization of the *d* orbitals is general and may have profound effect on the electronic and magnetic properties of other perovskite oxyhydrides. Below we will predict that SrFeO_2_H has an extremely high *T*_*N*_. With the cluster expansion approach, we predict that SrFeO_2_H takes the same ground state structure as that of SrCrO_2_H. This is reasonable since the ionic radius of the Fe^3+^ ion is close to that of the Cr^3+^ ion. The optimized lattice constants *a* and *c* for SrFeO_2_H are 3.997 Å and 3.645 Å, respectively. The computed phonon dispersion^26^ (see Supplementary Figure S4) indicates that SrFeO_2_H is dynamically stable. The spin exchange parameters calculated for the optimized SrFeO_2_H structures are listed in Table 1. SrFeO_2_H takes the G-type AFM order as the magnetic ground state since the NN AFM spin exchanges *J*_O_ (Fe-O-Fe) and *J*_H_ (Fe-H-Fe) are dominant. To our surprise, the spin exchange *J*_H_ (89.26 meV) in SrFeO_2_H is much stronger than *J*_O_ (39.63 meV) in SrFeO_2_H. Note that the much stronger Fe-H-Fe interaction is not mainly caused by the shorter Fe-H distance than the Fe-O distance, because similar results are also obtained in the cubic perovskite SrFeO_2_H structure. In fact, the much stronger Fe-H-Fe interaction is mainly because the out-of-plane Fe-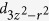 orbital is more delocalized than the in-plane Fe-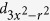 and 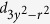 orbitals. Therefore, the σ bond between the H 1*s* orbital and Fe-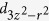 orbital is much stronger than the σ bond between the O-2*p* orbitals and the in-plane Fe 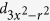/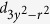 orbitals (see Supplementary Figure S6). Similar to the cases of SrCrO_2_H and SrVO_2_H, the weaker electrostatic potential of H^−^ ions exerting on the *d* electrons of Fe^3+^ ions leads to more delocalized Fe-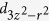 orbitals, which results in an anomalously strong spin exchange *J*_H_. Our MC simulations indicate that the *T*_*N*_ of SrFeO_2_H is around 826 K, which is even higher than that of BiFeO_3_ (*T*_*N*_ = 643 K)^35^ and SrFeO_2_ with a quasi-two-dimensional structure (*T*_*N*_ = 473 K)^36^,^37^. Our result suggests that the replacement of O^2−^ ions by H^−^ ions can enhance the magnetic interactions not only in *t*_2g_ *d*^2^ and *d*^3^ systems, but also in *d*^5^ systems. Our work suggests that the high magnetic transition temperature in LaSrCoO_3_H_0.7_^18^,^19^,^20^ and Sr_3_Co_2_O_4.33_H_0.84_^21^ should be also due to the H^−^ ion induced delocalization of the 3*d e*_*g*_ orbitals.

In summary, we perform a systematic theoretical study on the magnetic properties of perovskite oxyhydrides. The high magnetic transition temperature in SrCrO_2_H is revealed to be due to the delocalization of 3*d* orbitals in perovskite oxyhydrides. This is because H^−^ ions have weaker electronegativity and fewer electrons than O^2−^ ions. The more delocalized 3*d* orbitals in SrCrO_2_H make Cr-O-Cr superexchange strong and *T*_*N*_ high. This novel mechanism also applies to the case of SrVO_2_H. We predict that the σ-type Fe-H-Fe interactions in SrFeO_2_H are extraordinarily strong which also result from the delocalization of the 3*d* orbitals. The delocalization of *d* orbitals in oxyhydrides discovered in this work is universal and may also have profound effects on properties other than the magnetic properties.

## Methods

Our DFT calculations are performed on the basis of the projector augmented wave method^38^,^39^ encoded in the Vienna ab initio simulation package^40^,^41^ (VASP) using the generalized-gradient approximation (GGA) of Perdew, Burke, and Ernzerhof^42^. The plane-wave cutoff energy is set to be 450 eV. To properly describe the strong electron correlation in the 3*d* transition-metal oxide, the GGA plus on-site repulsion *U* method (GGA + *U*) is employed^43^. *U* = 4 eV and *J* = 1eV are applied to the 3*d* electron of Cr^3+^ ions. The maximally localized Wannier functions (MLWFs) are constructed with the Wannier90 program^44^,^45^. The spread functional is considered to be converged if the corresponding fractional change because two successive iterations is smaller than 10^−10^. To find the ground state structures of SrCrO_2_H, SrVO_2_H, and SrFeO_2_H, we adopt the cluster expansion approach^46^ by using the alloy theoretic automation toolkit (ATAT)^47^.

We perform parallel tempering Monte Carlo (PTMC) simulations^48^,^49^ to estimate the magnetic transition temperature. In PTMC simulations, many replicas with different temperature are simultaneously simulated and a virtual process exchanging configuration of these replicas is introduced. PTMC simulations can avoid a local minimum at low temperatures and can reduce relaxation time. We adopt a 10 × 10 × 10 supercell to perform PTMC simulations. Our test shows that the results obtained with a 12 × 12 × 12 supercell are almost the same as those with the 10 × 10 × 10 supercell. The number of replicas is set to 120.

## Additional Information

**How to cite this article**: Liu, K. *et al.* Orbital Delocalization and Enhancement of Magnetic Interactions in Perovskite Oxyhydrides. *Sci. Rep.* **6**, 19653; doi: 10.1038/srep19653 (2016).

## Supplementary Material

Supplementary Information

## Figures and Tables

**Figure 1:**
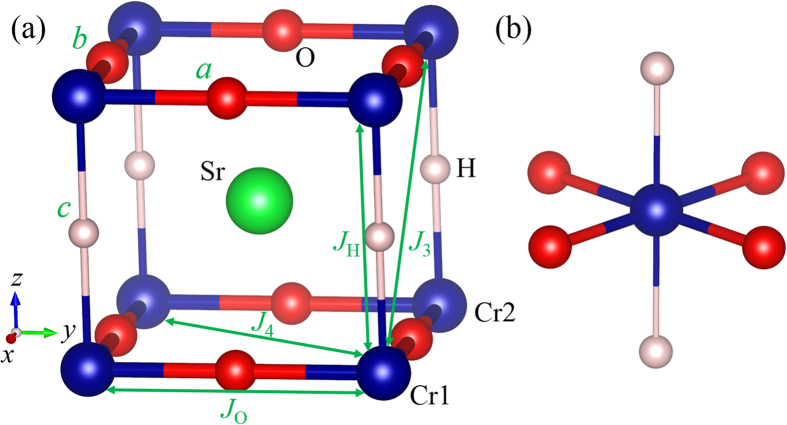
(**a**) Perspective view of the ground state structure of SrCrO_2_H. The green, blue, red, and greyish spheres represent the Sr^2+^, Cr^3+^, O^2−^, and H^−^ ions, respectively. The spin exchange paths *J*_O_, *J*_H_, *J*_3_, and *J*_4_ are also indicated. (**b**) The local structure of the CrO_4_H_2_ octahedron.

**Figure 2 f2:**
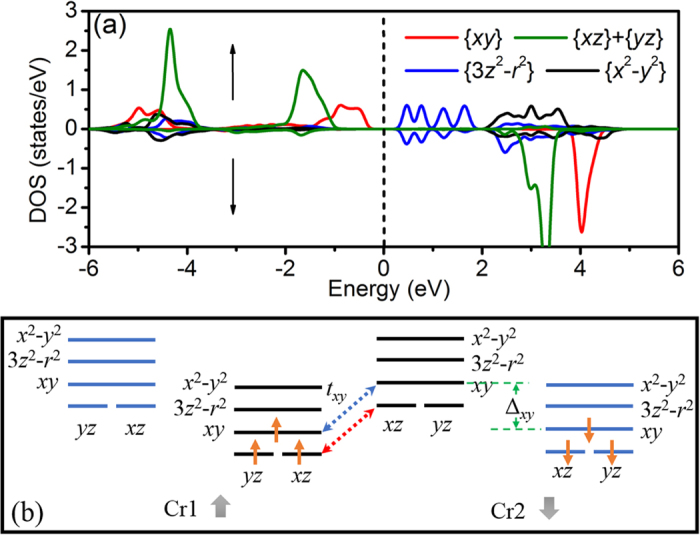
(**a**) PDOS of the spin-up Cr^3+^ ion in SrCrO_2_H with the G-type AFM order. (**b**) Energy level and electron occupation for the spin-up Cr1 ion and spin-down Cr2 ion. The positions of Cr1 and Cr2 are shown in Fig. 1a. The spin-up and spin-down levels are denoted by black and blue colors, respectively. The effective orbitals hoppings responsible for the Cr1-Cr2 superexchange interaction are illustrated.

**Figure 3:**
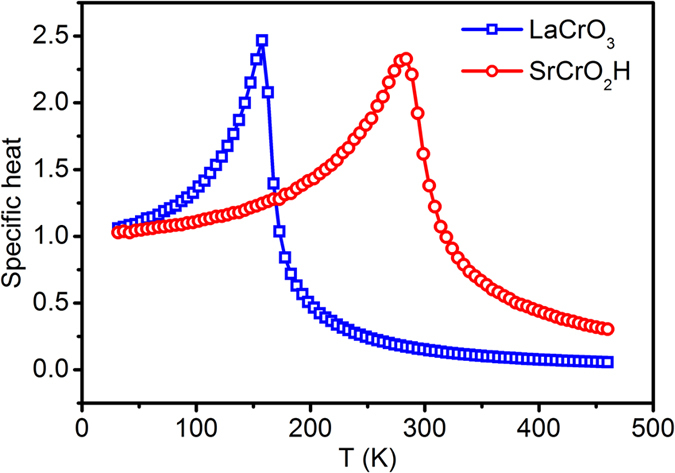
Specific heat of SrCrO_2_H and LaCrO_3_ calculated as a function of temperature from the MC simulations of the classical spin Hamiltonian.

**Figure 4:**
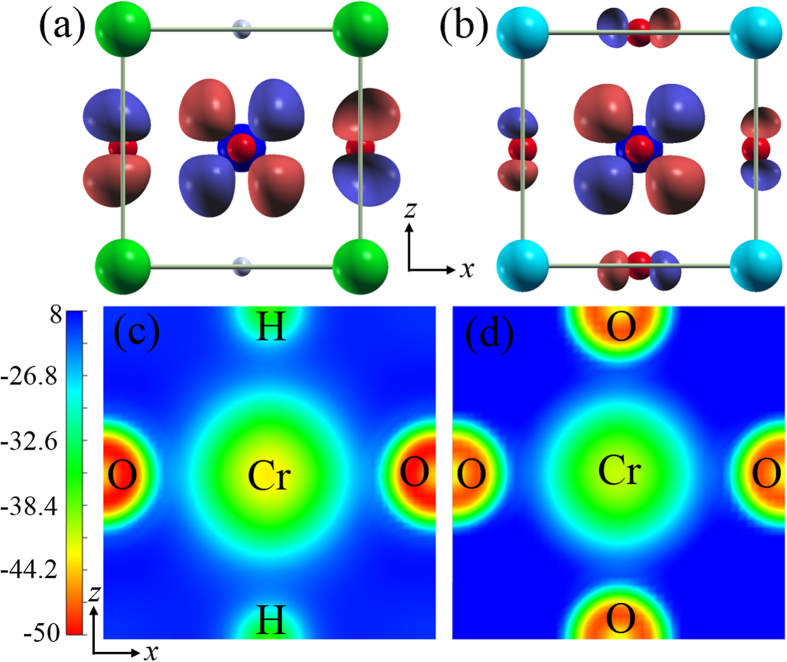
Isosurface plots of the Cr-*d*_*xz*_ like MLWFs for (a) cubic SrCrO_2_H and (b) cubic LaCrO_3_. The lobes on the O^2−^ ion in the MLWF of SrCrO_2_H are bigger than those of LaCrO_3_, indicating that the hybridization between Cr-*d*_*xz*_ orbital and O-*p*_*z*_ is stronger in SrCrO_2_H. Contour plots of the electrostatic potential in (**c**) cubic SrCrO_2_H and (**d**) cubic LaCrO_3_, projected on the *xz*-plane passing through Cr, H, and O sites. The electrostatic potential along the Cr-H direction is much weaker than that along the Cr-O direction in SrCrO_2_H.

**Table 1:** Spin exchange parameters and Neel temperature of perovskite systems considered in this work.

	*J*_O_ (meV)	*J*_H_ (meV)	*J*_3_ (meV)	*J*_4_ (meV)	*T*_*N*_ (K)
SrCrO_2_H (opt.)	23.95	3.26	−0.96	0.34	285
SrCrO_2_H (cubic)	29.69	1.27	−1.26	0.69	325
LaCrO_3_ (opt.)	8.88	–	–	0.38	137
LaCrO_3_ (cubic)	14.25	–	–	0.73	204
SrVO_2_H (opt.)	23.54	1.88	−0.71	0.17	255
SrFeO_2_H (opt.)	39.63	89.26	−4.42	4.22	950
SrFeO_2_H (cubic)	42.89	70.41	−3.89	−0.91	922

In SrCrO_2_H, SrVO_2_H, and SrFeO_2_H, the spin exchange paths *J*_O_, *J*_H_, *J*_3_, and *J*_4_ are defined in Fig. 1a. Positive (negative) values indicate that the spin exchange interactions are AFM (FM). The exchange parameters are effective by setting the spin magnitude to 1. In LaCrO_3_, *J*_O_ is the average NN spin exchange parameter and *J*_4_ is the average NNN spin exchange parameter. “opt.” refers to the structure optimized by GGA + *U* calculations, while “cubic” refers to the cubic perovskite structure.

**Table 2:** Effective hopping between the *d*_*xz*_, *d*_*yz*_, *d*_*xy*_ orbitals of nearest neighboring Cr ions in cubic SrCrO_2_H and LaCrO_3_ through the MLWF technique.

	*t*_*xz*_(eV)	*t*_*yz*_(eV)	*t*_*xy*_(eV)
SrCrO_2_H (cubic)	−0.385	0.002	−0.213
LaCrO_3_ (cubic)	−0.217	−0.017	−0.217
