# Supplementary material for: Orbital Delocalization and Enhancement of Magnetic Interactions in Perovskite Oxyhydrides
Source: Sci Rep. 2016 Jan 25;6:19653. doi: 10.1038/srep19653 (PMC4726173; doi:10.1038/srep19653)
Supplement: Supplementary Information [file srep19653-s1.doc]

**Supporting Information** **for**

**Orbital Delocalization and Enhancement of Magnetic Interactions**

**in Perovskite Oxyhydrides**

Kai Liu, Yusheng Hou, Xingao Gong, and Hongjun Xiang*

Key Laboratory of Computational Physical Sciences (Ministry of Education), State Key Laboratory of Surface Physics, Collaborative Innovation Center of Advanced Microstructures, and Department of Physics, Fudan University, Shanghai 200433, P. R. China

Email: [hxiang@fudan.edu.cn](mailto:hxiang@fudan.edu.cn)

1. **Properties of SrCrO_2_H with randomly distributed H^-^ ions**

Experiment showed H^-^ ions in SrCrO_2_H are randomly distributed in the O sites of ABO_3_ perovskite. Here, we show that the H^-^ ions should have a short-range order to some extent in the experimental SrCrO_2_H structure. We simulate the fully random SrCrO_2_H structure with the special quasirandom structure (SQS)^1,2^. Our calculation shows that the energy of the SQS structure is approximately 300 meV/Cr higher than that of the ground state. Note that there exists CrO_6-_*_x_*H*_x_* octahedron with *x ≠* 2 in the fully random structure. The presence of such octahedron makes the system highly unstable due to the Coulomb electrostatic interactions. The fully random structure has such a high energy so that the H^-^ ions may not be fully random. In the experimental cubic SrCrO_2_H structure, it appears to be more plausible that there exists only one kinds of CrO_6-_*_x_*H*_x_* octahedron, i.e., CrO_4_H_2_, while the orientation of these CrO_4_H_2_ octahedra is random.

To see the effect of H^-^ ion distribution on the magnetic properties, we investigate another SrCrO_2_H structure with a different orientation of the CrO_4_H_2_ octahedra. In this structure, some H-Cr-H bond angles are close to 90°, as show in Figure S1. The NN spin exchanges for the Cr-H-Cr path and for the Cr-O-Cr path is 0.63 meV and 24.41 meV, respectively. The MC simulation indicate that its *T_N_* is 275 K, close to that of the ground state. So the magnetic properties are very similar to those of the ground state. Thus, for simplicity we mainly discuss the results for the ground state of SrCrO_2_H in the main text.


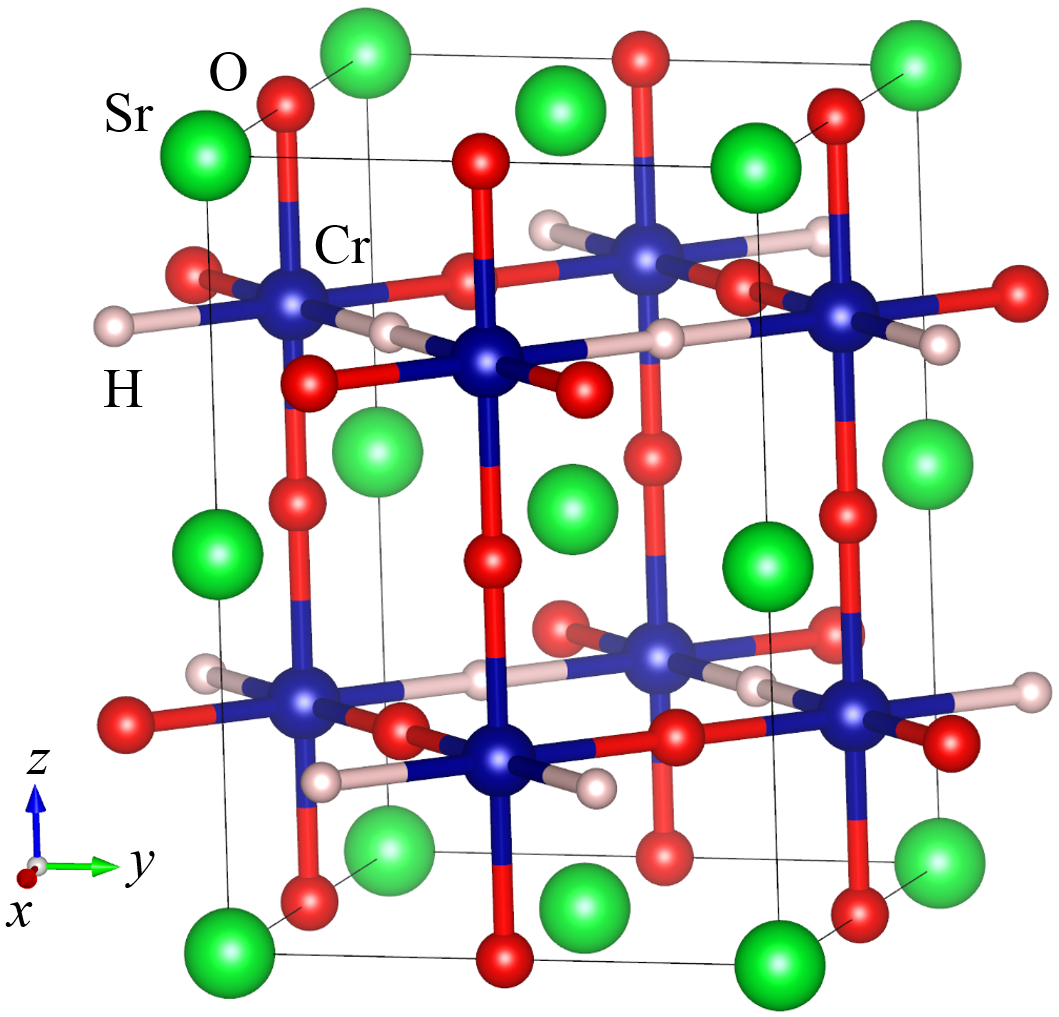


**Figure S1.** Another structure of SrCrO_2_H with corner-sharing CrO_4_H_2_ octahedra. Some H-Cr-H bond angles are close to 90°.

1. **Magnetic properties of SrVO_2_H**

Experiment show that SrVO_2_H adopts *P4/mmm* space group with lattice constant *a* = 3.93 Å and *c* = 3.66 Å.^3^ The lattice constants of our GGA + *U* optimized SrVO_2_H are *a* = 3.993 Å and *c* = 3.733 Å which are in good agreement with experiment. The 3d energy level of V^3+^ ion is shown in Figure S2. The obtained exchange parameters are summarized in Table I. Our MC simulations indicate that the *T_N_* in SrVO_2_H is around 208 K. From Table I, we note that spin exchange *J*_O_ is extremely large. Just like SrCrO_2_H, this results from the delocalization of the 3d orbitals induced by the H^-^ ions substitution.

**Table S1.** Neel temperature of considered perovskite systems estimated by PTMC simulations and mean field method. It can be seen that the mean-field approach generally overestimates the Neel temperature.

|  | PTMC simulation  *T_N_* (K) | Mean field method  *T_N_* (K) |
| --- | --- | --- |
| SrCrO_2_H (opt.) | 285 | 619 |
| SrCrO_2_H (cubic) | 325 | 735 |
| LaCrO_3_ (opt.) | 137 | 373 |
| LaCrO_3_ (cubic) | 204 | 608 |
| SrVO_2_H (opt.) | 255 | 719 |
| SrFeO_2_H (opt.) | 950 | 1726 |
| SrFeO_2_H (cubic) | 922 | 1503 |


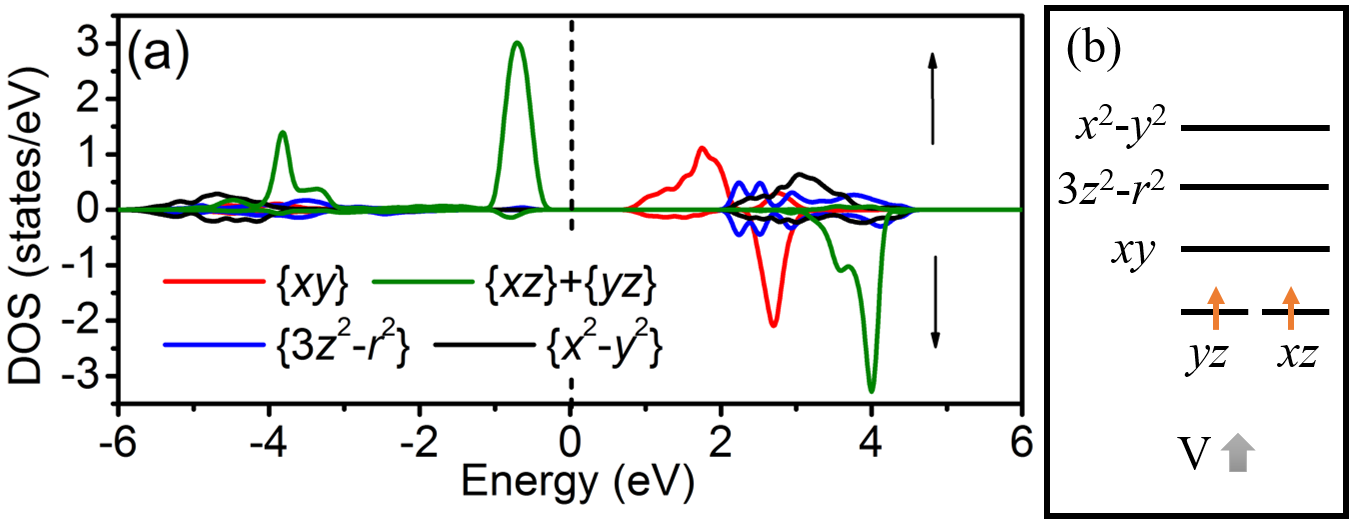


**Figure S2.** Electronic structure of the spin-up V^3+^ ion in SrVO_2_H with the G-type AFM order: a) PDOS plot. b) Energy level and electron occupation.


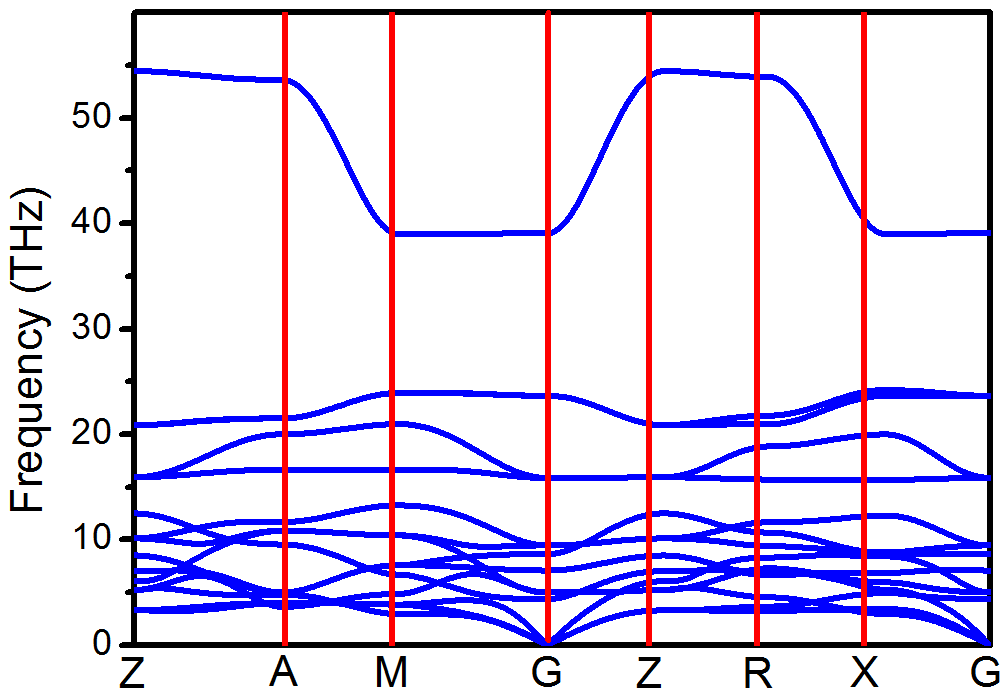


**Figure S3.** The phonon dispersions of the ground state structure of SrCrO_2_H computed by using the phonopy code^4^. There are no imaginary frequencies in the phonon dispersion, indicating its dynamical stability.


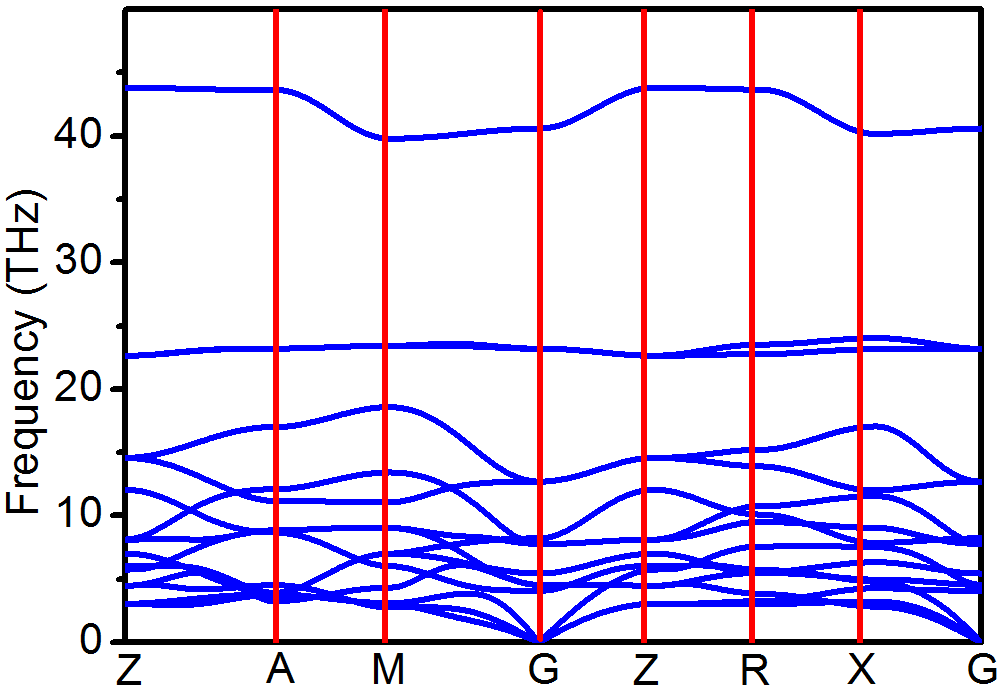


**Figure S4.** The phonon dispersions of optimized SrFeO_2_H. There are no imaginary frequencies in phonon dispersion, indicating its dynamical stability.


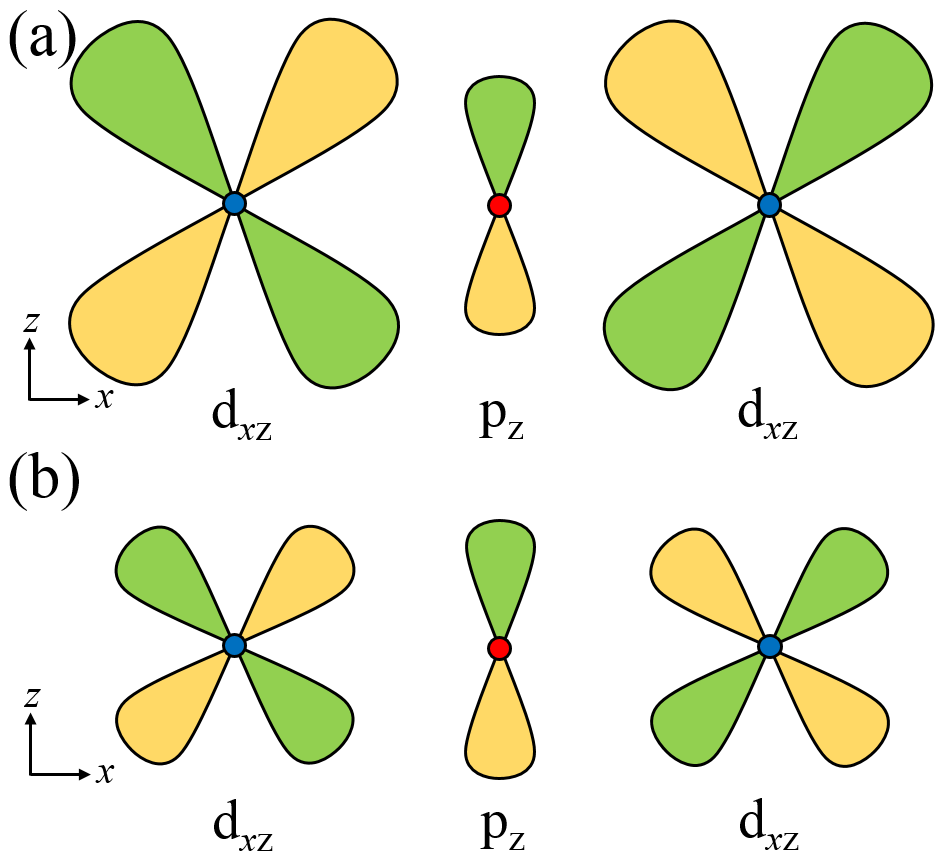


**Figure S5.** Illustration of the interaction between Cr-*d_xz_* and O-*p_z_* orbitals in (a) SrCrO_2_H and (b) LaCrO_3_. The blue and red cycle represent Cr^3+^ and O^2-^ ion, respectively. The lobe of *d_xz_* in SrCrO_2_H is bigger than that in LaCrO_3_, indicating that *d_xz_* in SrCrO_2_H is more delocalized than that in LaCrO_3_.


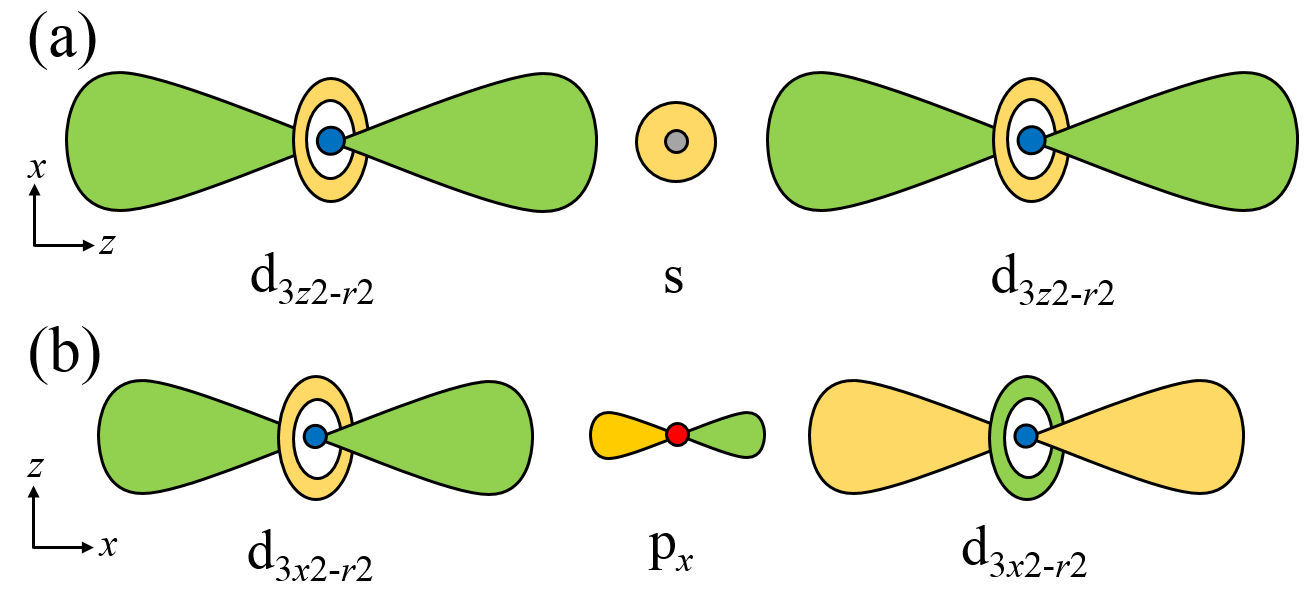


**Figure S6.** Illustration of the interactions (a) between Fe-*d*_3_*_z_*_2-_*_r_*_2_ and H-*s* orbitals, (b) between Fe-*d*_3_*_x_*_2-_*_r_*_2_ and O-*p_x_* orbitals in SrFeO_2_H. The blue, red, and gray cycle represent Fe^3+^, O^2-^ and H^-^ ion, respectively. The lobe of *d*_3_*_z_*_2-_*_r_*_2_ orbital is bigger than the lobes of *d*_3_*_x_*_2-_*_r_*_2_ orbitals, indicating that *d*_3_*_z_*_2-_*_r_*_2_ orbital along the Fe-H bond direction is more delocalized than that along the Fe-O bond direction.

**REFERENCE**

1. Wei, S. H., Ferreira, L. G., Bernard, J. E. & Zunger, A. Electronic Properties of Random Alloys: Special Quasirandom Structures. *Phys. Rev. B* **42**, 9622-9649 (1990).

2. Zunger, A., Wei, S. H., Ferreira, L. G. & Bernard, J. E. Special Quasirandom Structures. *Phys. Rev. Lett.* **65**, 353-356 (1990).

3. Romero, F. D. et al. Strontium Vanadium Oxide-Hydrides: "Square-Planar" Two-Electron Phases. *Angew. Chem. Int. Ed.* **53**, 7556-7559 (2014).

4. Togo, A., Oba, F. & Tanaka, I. First-principles Calculations of the Ferroelastic Transition between Rutile-type and CaCl_2_-type SiO_2_ at High Pressures. *Phys. Rev. B* **78**, 134106 (2008).
